# Trends in research on preterm birth in twin pregnancy based on bibliometrics

**DOI:** 10.1515/med-2025-1202

**Published:** 2025-05-17

**Authors:** Biaobiao Wang, Weishe Zhang, Xiuqing Lv, Qi Li, Jingrui Huang

**Affiliations:** Department of Obstetrics, Xiangya Hospital Central South University, Changsha, 410008, China; Hunan Engineering Research Center of Early Life Development and Disease Prevention, Changsha, China; Reproductive Medicine Center, Xiangya Hospital Central South University, Changsha, China

**Keywords:** bibliometric analysis, twin pregnancy, preterm birth

## Abstract

**Background:**

Twin pregnancies are associated with a higher risk of perinatal mortality compared to singleton pregnancies. This study evaluated the developmental trends and summarized the key features of research on preterm births in twin pregnancies.

**Methods:**

A bibliometric analysis was conducted using publications on preterm births in twin pregnancies from 2014 to 2023, retrieved from the Web of Science Core Collection database. Network and visual analyses were performed using VOSviewer and CiteSpace software. In total, 1,378 articles were included.

**Results:**

The number of publications in this field has shown a steady increase over the past decade. The United States, China, and England collectively contributed more than half of all publications. King’s College London, Columbia University, and Jefferson University were identified as the most influential institutions, fostering extensive collaboration and academic exchange. *Am J Obstet Gynecol* emerged as the most cited journal. Research has predominantly focused on clinical practices, including prevention strategies, risk factor identification, and perinatal outcomes.

**Conclusion:**

This bibliometric analysis offers a comprehensive overview of research trends on preterm births in twin pregnancies, highlighting major contributors, influential institutions, and primary research foci. The findings provide valuable insights for researchers, aiding the identification of future research directions in this critical field.

## 1 Introduction

The incidence of twin pregnancies has increased in recent years, largely due to the growing use of assisted reproductive technology (ART) and the increasing maternal age at conception. ART is associated with a higher likelihood of twin or triplet pregnancies, and advanced maternal age is also related to an increased probability of multiple pregnancies [1]. In addition, older maternal age, often accompanied by ovarian and uterine aging, can result in poor reproductive performance, necessitating the use of ART [2]. Twin pregnancies are associated with an elevated risk of pregnancy complications, including preterm birth, preeclampsia, intrauterine growth restriction, and other adverse outcomes [3].

Monochorionic twin pregnancies, which involve a shared placenta, carry an even higher risk of complications, such as twin-to-twin transfusion syndrome and twin anemia-polycythemia sequence [4]. Furthermore, compared to singletons, neonates from twin pregnancies face an increased risk of adverse outcomes, including congenital anomalies, cerebral palsy, intrauterine growth restriction, and stillbirth [5]. Although twin pregnancies account for only about 3% of all live births, they represent up to 15% of admissions to special care units [6,7].

Twin pregnancies are also associated with a higher risk of perinatal mortality, predominantly due to preterm birth [8]. The preterm birth rate in twin pregnancies has significantly increased over the past three decades, paralleling the global increase in twin birth rates. While 8.76% of women with singleton pregnancies deliver before 37 weeks of gestation and 2.20% before 34 weeks, the corresponding rates for twin pregnancies are markedly higher, being 62.05 and 20.28%, respectively [7].

Given these heightened risks, it is essential to summarize the characteristics and research topics related to preterm birth in twin pregnancies. Bibliometric analysis is a valuable statistical tool for analyzing published literature and constructing relationship maps, providing insights into development trends and research hotspots [9].

In this study, we conducted a bibliometric analysis of the current trends in preterm birth among twin pregnancies. We identified and analyzed documents published over the past decade, focusing on the number of publications per year, contributing authors, affiliated countries, journals, institutions, references, and keywords. Our results provide a comprehensive overview of the current state of research, highlight development trends, identify research hotspots, and outline potential directions for future studies.

## 2 Methods

### 2.1 Data source and search strategy

**Figure 1:**
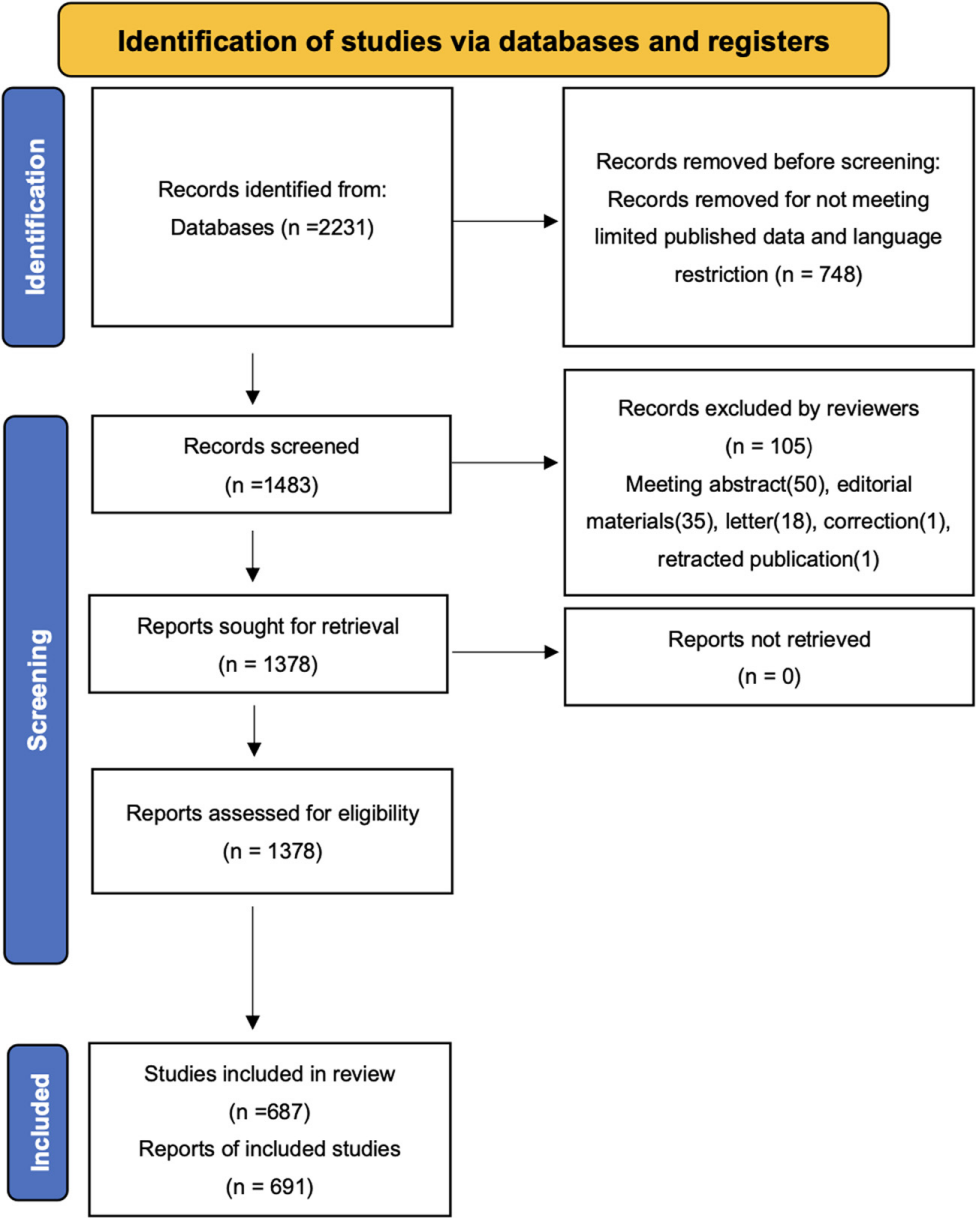
PRISMA flow diagram of the identification of studies via the database.

The Web of Science Core Collection, a leading citation database of scientific publications, including scholarly journals and citation index information, served as the primary data source. The search strategy is illustrated in Figure 1. In total, 2,231 records were initially identified in the database. After applying restrictions to include only English language publications between January 1, 2014, and December 1, 2023, 1,483 records remained. Subsequently, 105 records, including meeting abstracts (50), editorial materials (35), letters (18), corrections (1), and retracted publications (1), were excluded, resulting in 1,378 documents that were exported for analysis.

### 2.2 Data analysis

Following the data search, information on publication years, affiliated countries, institutions, authors, journals, and references was collected for further analysis and discussion [10]. CiteSpace and VOSviewer were utilized as the primary tools for bibliometric analysis. CiteSpace was used for cocitation analysis and citation burst detection, while VOSviewer was used for network visualization and cluster analyses, including coauthorship analysis, journal citations, reference citations, and keyword cooccurrence analysis [11].

Each node in the distribution map represented a country, institution, author, journal, or reference. In CiteSpace, a purple ring around a node indicated centrality, signifying the occurrence of a pivotal node within the field and its substantial influence. Centrality acted as a hinge point, with nodes representing countries or institutions with higher centrality if they were linked to multiple other nodes. Lines between nodes represent cooperative relationships, with the thickness of the lines denoting the strength of collaboration [10]. The annual and cumulative publications of institutions were visualized using bar charts.

## 3 Results

### 3.1 Annual publications on preterm birth in twin pregnancies

**Figure 2:**
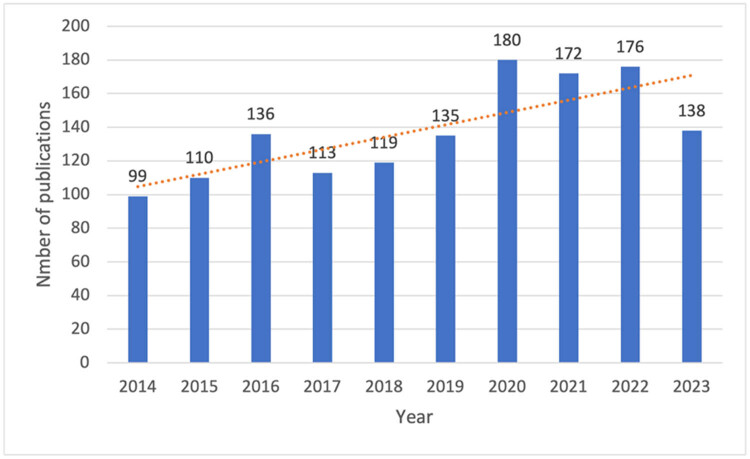
Annual publications on preterm births in twin pregnancies.

The number of publications reflects trends over time, representing public interest in a specific field. Figure 2 illustrates the annual distribution of publications on preterm births in twin pregnancies from 2014 to 2023. Over the past decade, the number of publications has exhibited a steady upward trend, peaking at 180 papers in 2020. However, a slight decline was observed from 2021 to 2023, likely attributable to time restrictions in data collection, as some 2023 publications were not included. Overall, the growth in publication numbers highlighted increasing attention to preterm births in twin pregnancies.

### 3.2 Geographical distribution of published countries

**Figure 3:**
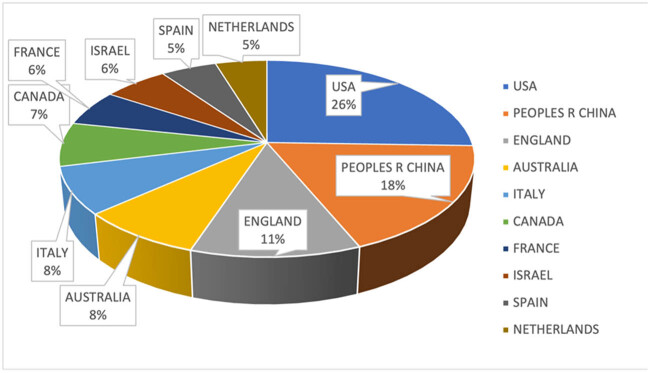
Top ten countries by number of publications.

In all, 93 countries contributed to research in this field. The top 10 were the USA, China, England, Australia, Italy, Canada, France, Israel, Spain, and the Netherlands. The USA, China, and England collectively accounted for more than half of all publications. Detailed percentages of publications from the top 10 countries are presented in Figure 3.

**Figure 4:**
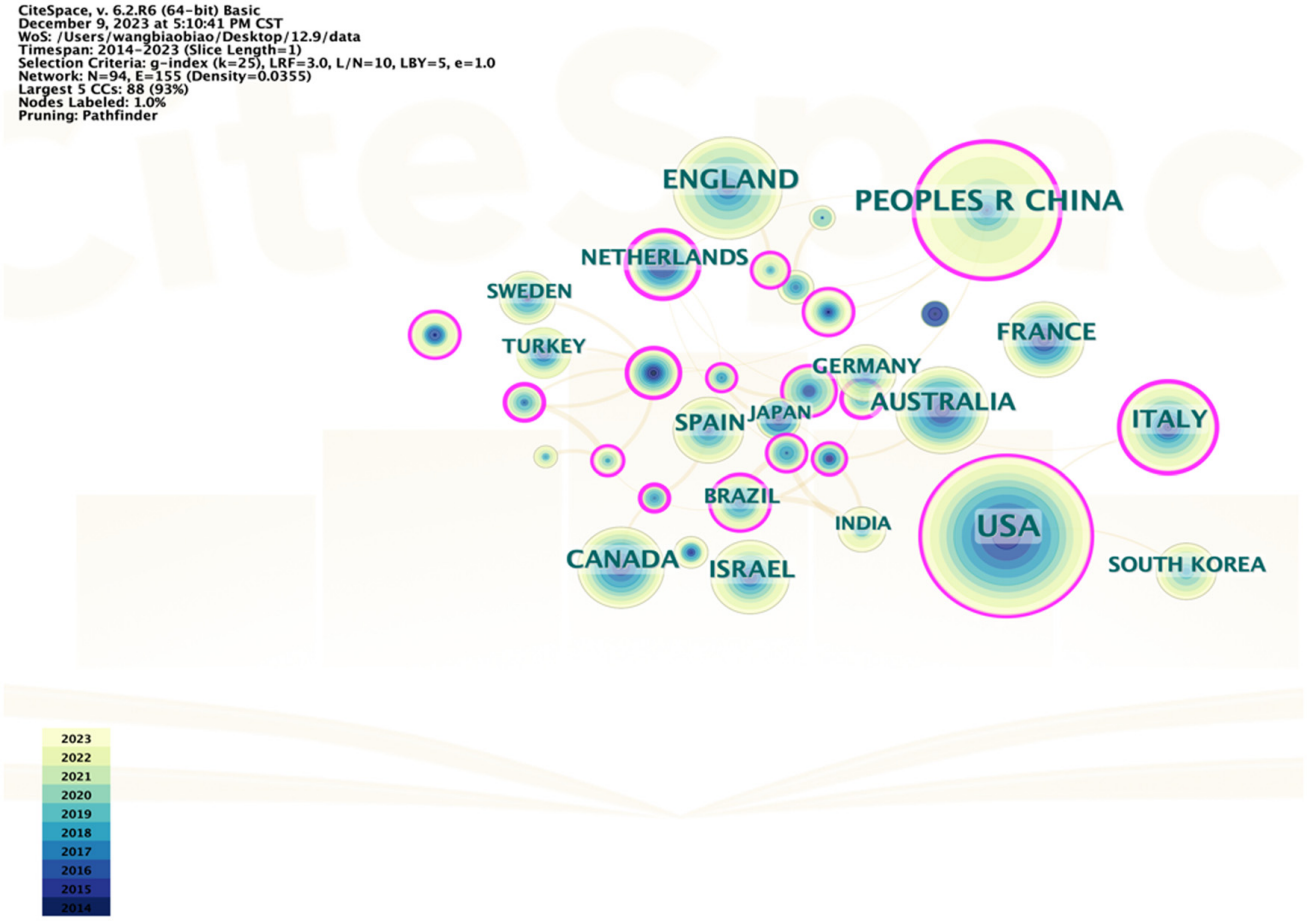
Coauthorship among countries with at least 15 publications. Nodes represent countries, with blue and purple rings indicating publication counts and centrality, respectively.

Using CiteSpace, we visualized the geographical distribution and cooperative networks among countries (Figure 4). The blue and yellow color ring represents the proportional articles published in a certain year. The purple ring outside the yellow ring represents the centrality which indicates the influence and importance of the research. Countries with the highest number of publications such as the USA, and China are labeled with purple rings, indicating that these countries maintain high research interest in this field and play an important role in the development of research of the preterm birth in twin pregnancy. Interestingly, although Finland and Italy were not among the top ten countries by publication count, they ranked in the top five for centrality, demonstrating that research impact is not solely determined by the number of publications.

### 3.3 Institutional coauthorship

**Figure 5:**
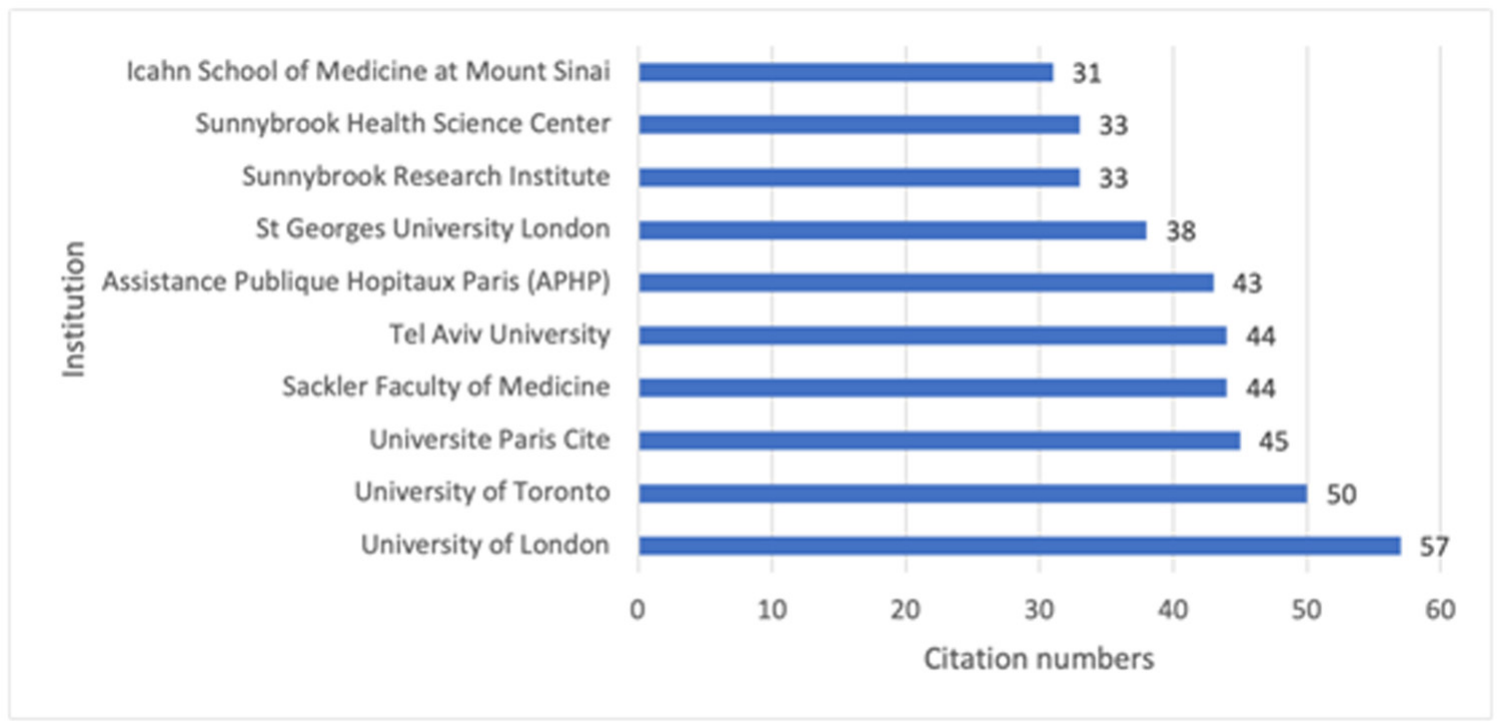
Top ten institutions by publication count.

In total, 2,046 institutions published papers related to preterm births in twin pregnancies. The top ten institutions contributing the most publications are shown in Figure 5. Among these, the University of London, the University of Toronto, and the Université Paris Cité led by a significant margin. Rankings based on centrality are presented in Table 1, showing King’s College London, Columbia University, and Jefferson University as the top three central institutions in this field.

**Table 1:** Top ten institutions ranked by centrality in studies on preterm births in twin pregnancies

Rank	Centrality	Institute
1	0.64	King’s College London
2	0.42	Columbia University
3	0.40	Jefferson University
4	0.39	Catholic University of the Sacred Heart
5	0.39	University of London
6	0.34	Guy’s & St Thomas’ NHS Foundation Trust
7	0.32	Icahn School of Medicine at Mount Sinai
8	0.25	King’s College Hospital NHS Foundation Trust
9	0.25	King’s College Hospital
10	0.25	Michigan State University

**Figure 6:**
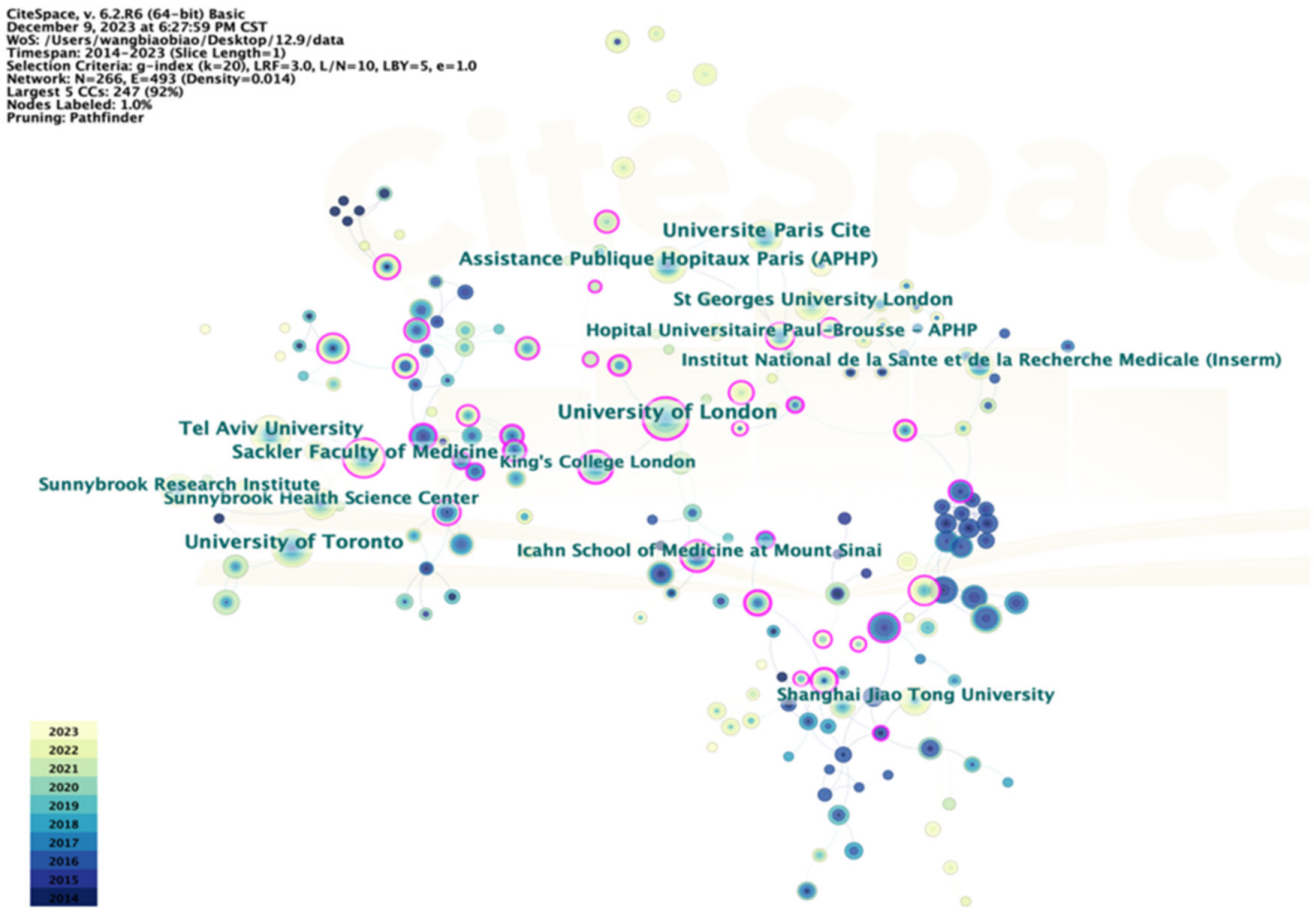
Collaboration network among institutions. Nodes represent institutions, with blue and purple rings denoting publication counts and centrality, respectively.

A coauthorship visual analysis was performed, setting the minimum publication threshold per institution to 20. Active collaborations were observed among institutions worldwide (Figure 6). King’s College London demonstrated the highest centrality, maintaining strong connections with 11 other institutions, including the University of London, the University of Bristol, and Guy’s and St Thomas’ NHS Foundation Trust.

### 3.4 Analysis of coauthorship and cocitation

**Figure 7:**
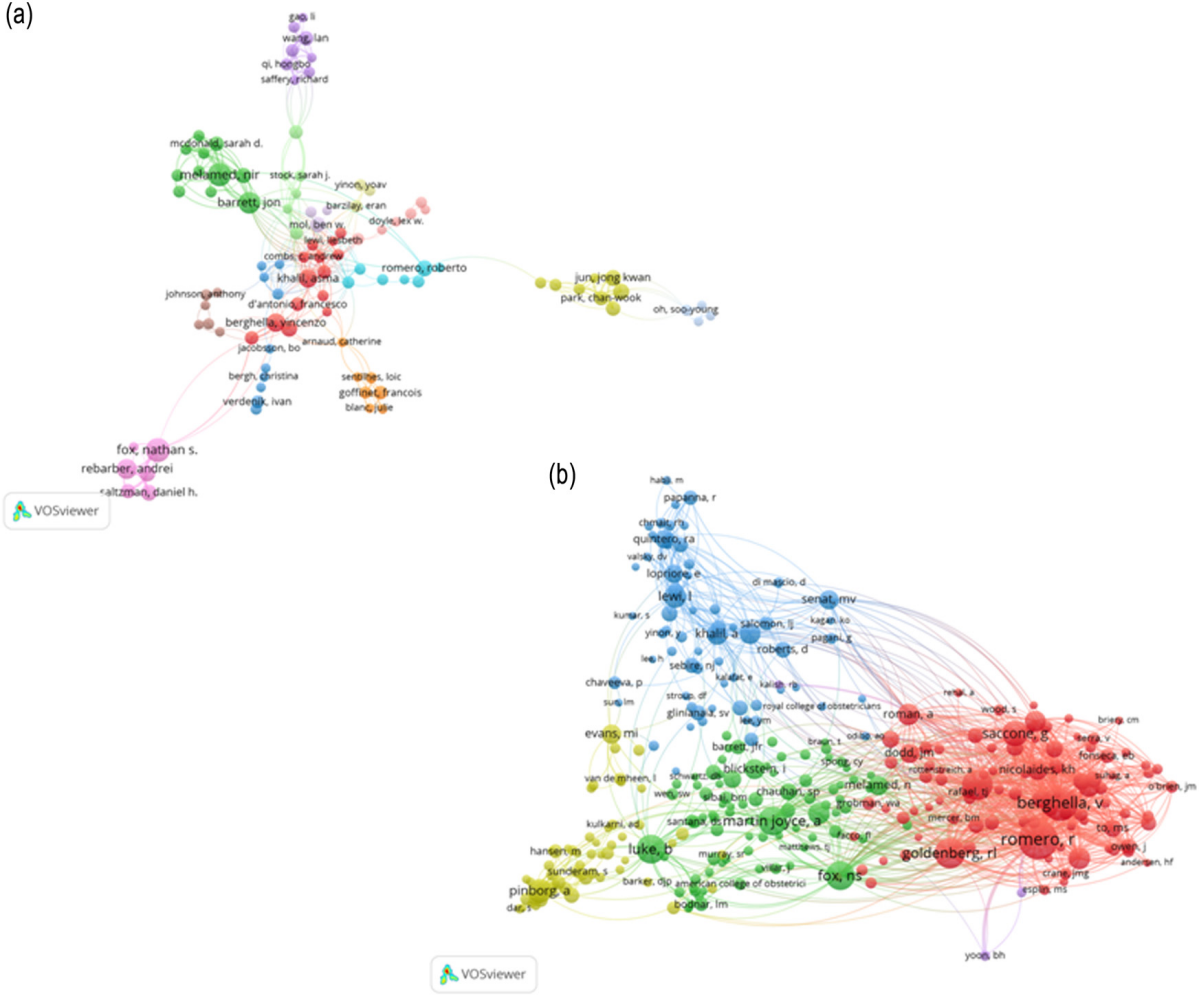
Visualization of coauthorship and cocitation analyses. (a) Coauthorship of 146 authors with at least 5 publications, represented in 14 clusters. (b) Cocitation analysis of 309 authors cited at least 20 times, categorized into 5 clusters.

The 1,378 documents analyzed included contributions from 7,087 authors, of whom 146 had authored at least 5 papers. The results of coauthorship analysis conducted using VOSviewer is shown in Figure 7a. This analysis organized 129 authors into 14 clusters, each represented by a distinct color corresponding to a research team. The top ten most prolific authors, with no fewer than ten publications, are listed in Table 2.

**Table 2:** Top ten authors ranked by publication and cocitation counts

Rank	Author	Publications	Co-cited author	Co-citations
1	Fox, NS.	28	Romero, R.	457
2	Melamed, N.	26	Berghella, V.	369
3	Barrett, J.	23	Conde-Agudelo, A.	276
4	Khalil, A.	22	Goldenberg, RL.	275
5	Rebarber, A.	19	Luke, B.	250
6	Berghella, V.	17	Martin, JA.	247
7	Romero, R.	13	Fox, NS.	246
8	Saccone, G.	13	Saccone, G.	201
9	Mol, Ben W.	13	Pinborg, A.	176
10	Saltzman, DH.	13	Lewi, L.	155

Cocitation analysis was used to evaluate instances where authors, journals, or references of two or more papers were cited by the same article. The top ten authors based on cocitation counts are also presented in Table 2. For visualization, 309 authors cocited at least 20 times were selected. Five clusters were identified, with the largest including 94 authors, followed by clusters of 85, 65, 62, and 3 authors (Figure 7b).

### 3.5 Top ten cited and cocited journals

The 1,378 publications were distributed across 349 journals. Table 3 lists the top ten cited and cocited journals in this field. The *American Journal of Obstetrics and Gynecology* ranked first with 2,454 citations, followed by *Ultrasound in Obstetrics and Gynecology* (1,483 citations) and *Fertility and Sterility* (655 citations).

**Table 3:** Top ten cited and cocited journals in studies on preterm births in twin pregnancies

Rank	Journal	Counts	Co-cited journal	Counts
1	Am J Obstet Gynecol	2,454	Am J Obstet Gynecol	6,299
2	Ultrasound Obstet Gynecol	1,483	Obstet Gynecol	3,092
3	Fertil Steril	1,010	Ultrasound Obstet Gynecol	2,709
4	Hum Reprod	891	Hum Reprod	1,676
5	J Matern Fetal Neonatal Med	867	Fertil Steril	1,617
6	Obstet Gynecol	708	BJOG: an international journal of obstetrics and gynecology	1,407
7	BJOG: an international journal of obstetrics and gynecology	607	J Matern Fetal Neonatal Med	1,374
8	PLoS One	591	Lancet	1,169
9	MMWR Surveillance Summaries	530	N Engl J Med	974
10	Eur J Obstet Gynecol Reprod Biol	487	Eur J Obstet Gynecol Reprod Biol	834

**Figure 8:**
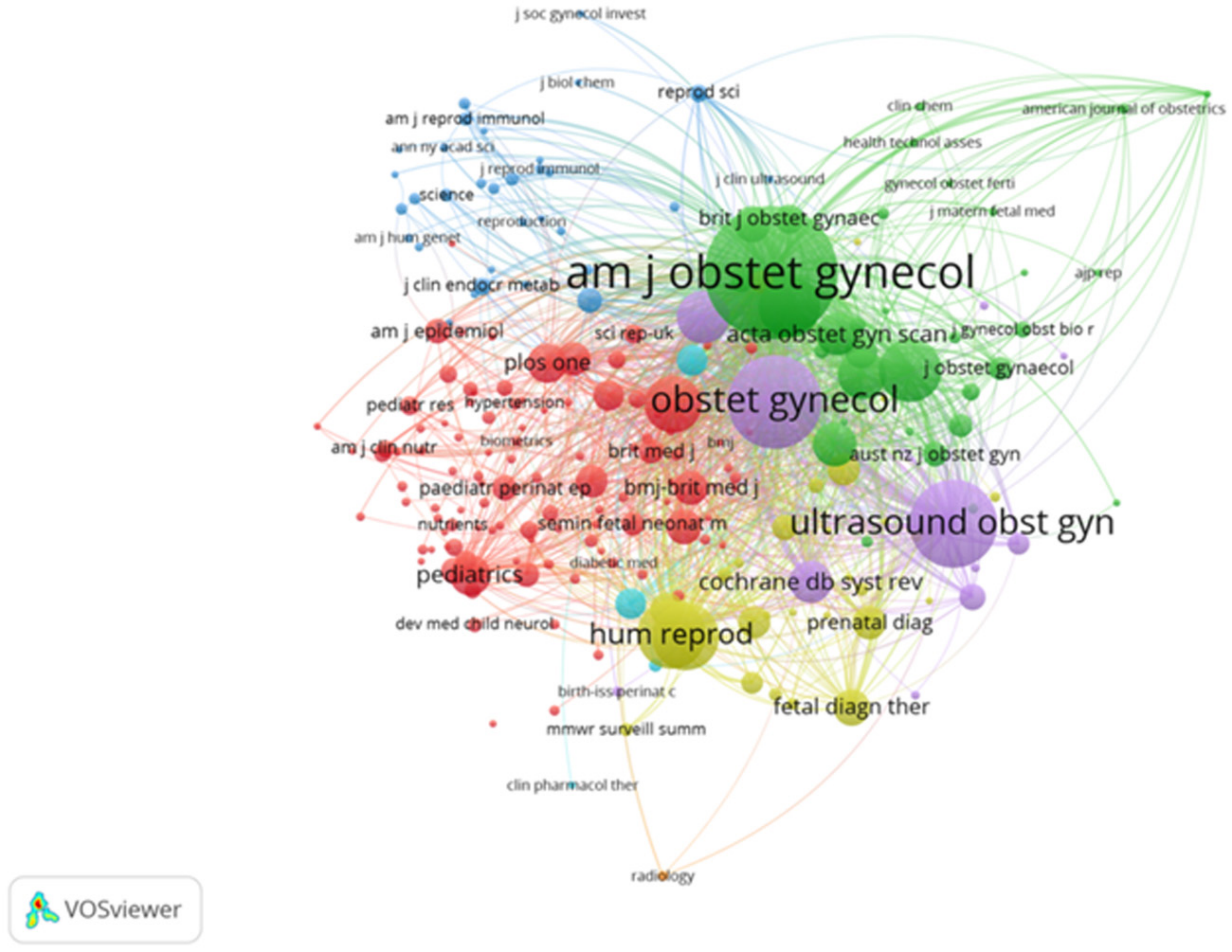
Cocitation map of journals with a threshold of 20 citations, divided into 7 clusters by color.

Cocitation analysis was performed on 217 journals with at least 20 citations, generating a network map divided into 7 clusters (Figure 8). The top three cocited journals were the *American Journal of Obstetrics and Gynecology* (6,299 cocitations), *Obstetrics and Gynecology* (3,092), and *Ultrasound in Obstetrics and Gynecology* (2,709).

### 3.6 Highly cited and cocited references

**Figure 9:**
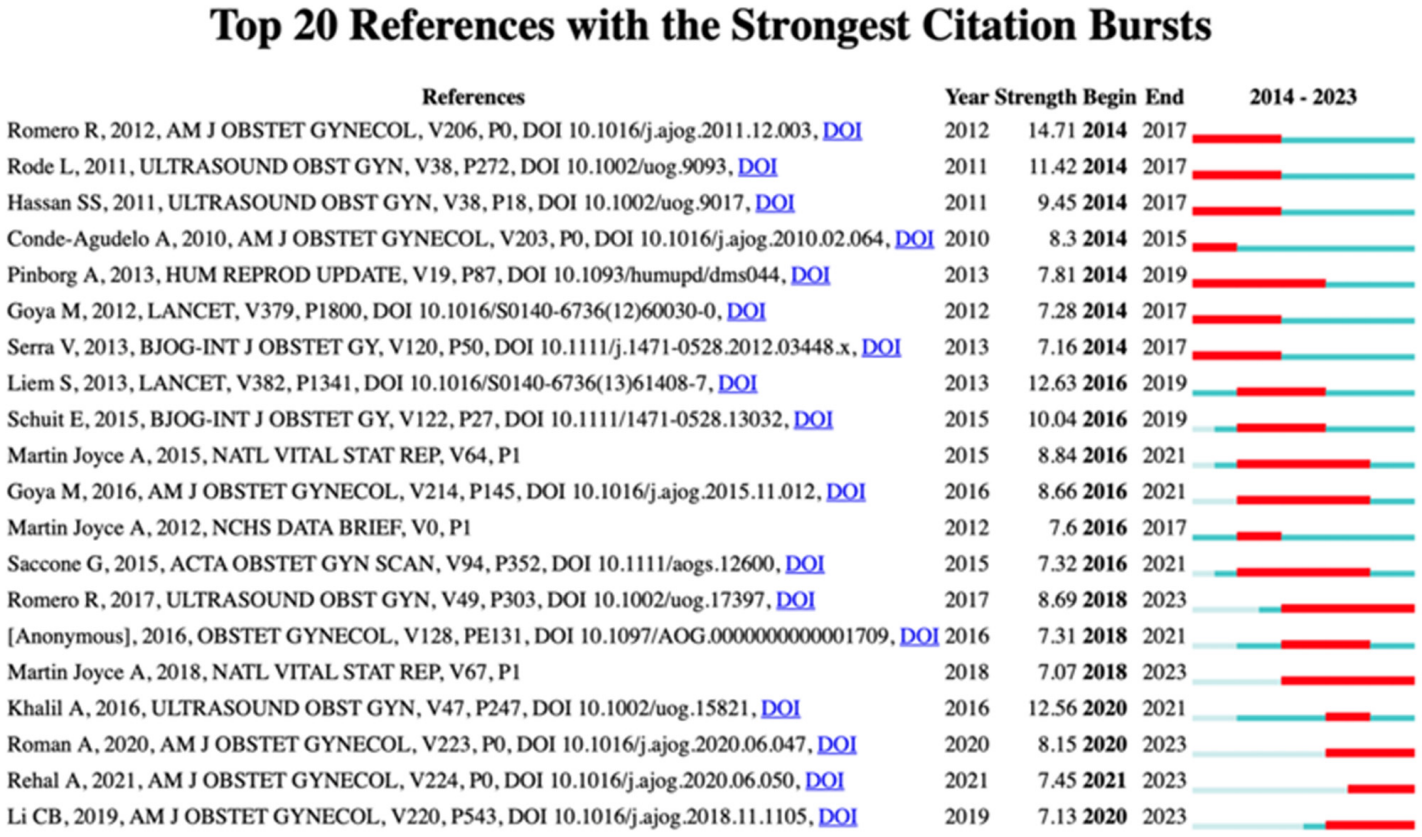
Top 20 references with the strongest citation bursts by year.

The frequency of citations reflects the influence and interest of an article within the research community. In all, 167 cocited references were included in the VOSviewer analysis. Figure 9 illustrates the top 20 references with the strongest citation bursts by year. The reference with the strongest citation burst, published in 2012, was titled “Vaginal progesterone in women with an asymptomatic sonographic short cervix in the mid-trimester decreased preterm delivery and neonatal morbidity: A systematic review and meta-analysis of individual patient data.” The second highest, published in 2013, was “Cervical pessaries for the prevention of preterm birth in women with multiple pregnancies.”

**Figure 10:**
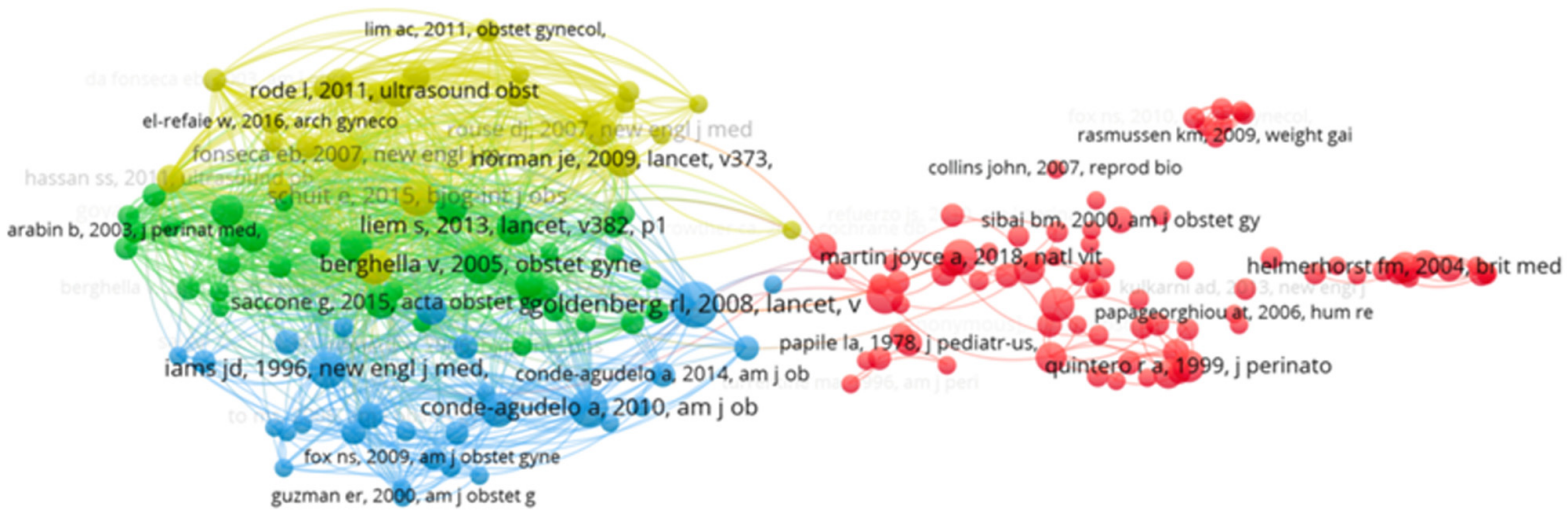
Cocitation map of references, where nodes represent individual references, and colors indicate clusters.

Four clusters of cocitation analysis were identified using VOSviewer (Figure 10). Larger nodes in the visualization represent references with higher cocitation frequencies. The clusters, differentiated by colors, represent 78 (red), 35 (green), 29 (blue), and 25 (yellow) references, respectively. The top ten cited references are listed in Table 4. The most frequently cited paper, “Cohort Profile Update: The Norwegian Mother and Child Cohort Study,” published in the *International Journal of Epidemiology* in 2016, was cited 505 times. The second, titled “ISUOG Practice Guidelines: The role of ultrasound in twin pregnancy,” had 314 citations. The third, “Coronavirus in pregnancy and delivery: rapid review,” explored the impact of coronavirus on pregnancy and delivery. Notably, two of the top ten references were published in the *American Journal of Obstetrics and Gynecology*.

**Table 4:** Top ten cited papers on preterm births in twin pregnancies

Rank	Title	First author	Cited frequency	Journal	Year
1	Cohort profile update: the Norwegian mother and child cohort study (MoBa)	Magnus, P.	505	Int J Epidemiol	2016
2	ISUOG Practice Guidelines: role of ultrasound in twin pregnancy	Khalil, A.	314	Ultrasound Obstet Gynecol	2016
3	Coronavirus in pregnancy and delivery: rapid review	Mullins, E.	274	Ultrasound Obstet Gynecol	2020
4	Vaginal progesterone for preventing preterm birth and adverse perinatal outcomes in singleton gestations with a short cervix: a meta-analysis of individual patient data	Roberto, R.	262	Am J Obstet Gynecol	2018
5	Maternal and fetal genetic effects on birth weight and their relevance to cardio-metabolic risk factors	Warrington, NM.	261	Nat Genet	2019
6	The risk factors for postpartum depression: A population-based study	Silverman, ME.	211	Depress Anxiety	2017
7	Systematic review of worldwide trends in ART 2004–2013	Kushnir, VA.	190	Reprod Biol Endocrinol	2017
8	The role of routine cervical length screening in selected high- and low-risk women for preterm birth prevention	McIntosh, JJ.	140	Am J Obstet Gynecol	2016
9	Ursodeoxycholic acid vs placebo in women with intrahepatic cholestasis of pregnancy (PITCHES): a randomized controlled trial	Chappell, LC.	138	Lancet	2019
10	ART Surveillance – United States, 2012	Sunderam, S.	136	MMWR Surveillance Summaries	2015

### 3.7 Keyword analysis

**Figure 11:**
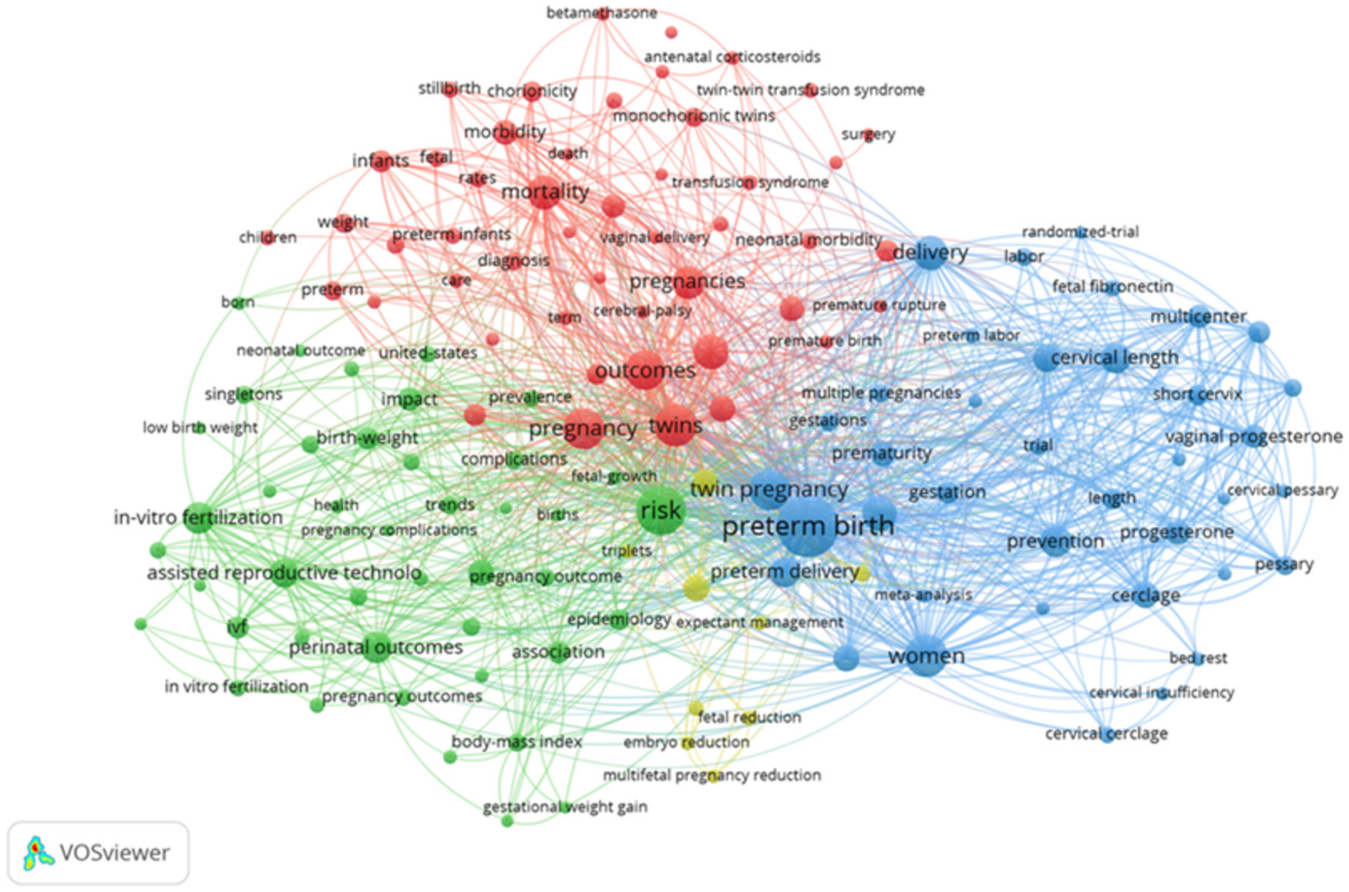
Cooccurrence network of keywords with a minimum frequency of 20. Node sizes indicate keyword frequency, and lines represent connections between keywords. Colors indicate different clusters.

Keywords offer insights into current research trends and directions for future studies. A cooccurrence network was constructed using 138 keywords with at least 20 occurrences (Figure 11). The keywords were grouped into four clusters. Cluster 1 focused on interventions for preterm births, including terms, such as “cervical length,” “cerclage,” and “vaginal progesterone.” Cluster 2 highlighted risks associated with preterm births in twin pregnancies, with keywords including “ART,” “pregnancy complications,” and “body mass index.” Cluster 3 involved outcomes of preterm births in twin pregnancies, using terms such as “morbidity” and “mortality.” Cluster 4 included additional topics relevant to twin pregnancies and preterm birth.

**Figure 12:**
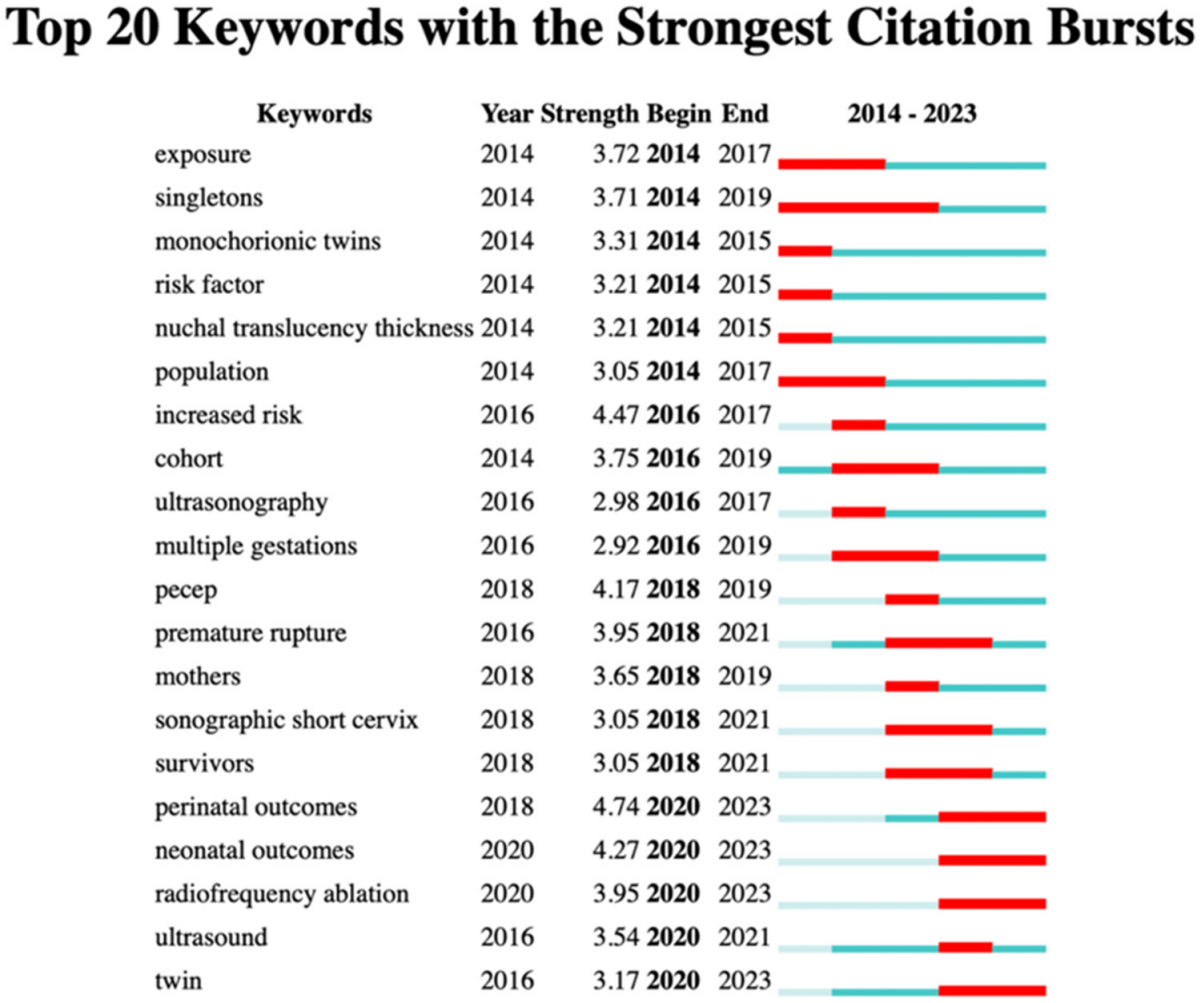
Top 20 keywords with the strongest citation bursts by year.

The top 20 keywords with the strongest citation bursts from 2014 to 2023 are shown in Figure 12. The keyword “perinatal outcome” emerged in 2020 and remains a focus to date. Similarly, “neonatal outcome,” which also appeared in 2020, indicated the sustained interest in outcomes of preterm births in twin pregnancies.

## 4 Discussion

Twin pregnancies have emerged as a significant focus of research due to the increasing prevalence attributed to advancements in ART and the increasing maternal age at conception [3]. This trend has brought a concomitant increase in twin-related complications and comorbidities, necessitating distinct management strategies compared to singleton pregnancies [12,13]. For example, low-molecular-weight heparin has demonstrated efficacy in preventing preeclampsia, treating fetal growth restriction, and reducing the risk of venous thromboembolism during twin pregnancies [14]. However, the relationship between inherited thrombophilia and adverse thrombotic pregnancy outcomes remains uncertain [15], highlighting the need for further exploration of its application in twin pregnancies [16]. These gaps suggest the substantial scope for research on optimizing maternal and fetal care in twin pregnancies and the related complications [5].

From 2014 to 2023, the number of publications on twin pregnancies steadily increased. The US, as the leading contributor, hosts many of the top publishing institutions, reflecting its pivotal role in advancing the field. China and England also ranked among the top three countries by publication volume, forming collaborative networks with other nations and institutions. These findings demonstrate the global commitment for investigating preterm births in twin pregnancies.

Among the top ten cited and cocited journals, the *American Journal of Obstetrics and Gynecology* holds the highest rank, highlighting its critical role as a platform for disseminating impactful research in obstetrics and gynecology. This dominance indicates the journal’s relevance for future submissions in this field.

Despite the growing body of literature, studies specifically addressing preterm births in twin pregnancies remain a relatively small proportion of the overall research landscape. Clinical practice, particularly prevention, is a cornerstone of this field. For instance, studies on vaginal progesterone for women with a short cervix during the mid-trimester [17] and the use of cervical pessaries in multiple pregnancies [18] have shown promise in reducing preterm delivery and neonatal morbidity. These interventions exemplify how research findings can translate into effective clinical strategies, with progesterone and pessaries now widely recognized as preventive measures in twin pregnancies [3,6].

The diversity of research approaches, spanning epidemiological studies, guidelines, reviews, and clinical trials, reflects the multifaceted nature of the field. While the ultimate goal is to comprehensively understand preterm births in twin pregnancies and guide clinical practice, many aspects remain enigmatic, necessitating further investigation.

Keyword analysis provided insight into evolving research priorities [19]. “Perinatal outcome” has shown the strongest citation burst in recent years, maintaining a consistent upward trend [20,21]. Keywords, such as “increased risk,” emphasize the importance of identifying risk factors [8,22], while “short cervix” highlights the significance of cervical incompetence as a critical issue in twin pregnancies [23,24]. These findings indicate that clinical practice remains the dominant research focus, with ongoing efforts needed to address unresolved questions in the field.

## 5 Conclusion

This study provides a comprehensive overview of research trends on preterm births in twin pregnancies, analyzing contributions from countries, institutions, authors, journals, and references from 2014 to 2023. The data revealed an upward trend in publications, with the United States, China, and England emerging as leading contributors. Global collaboration networks among countries, institutions, and authors have been firmly established. Research in this area has primarily focused on clinical practice, including prevention strategies, risk factor identification, and perinatal outcomes. These findings offer valuable insights into past advancements and outline directions for future exploration, emphasizing the importance of continued efforts to address the complexities of preterm births in twin pregnancies.
